# Pulse Rate Variability Analysis Using Remote Photoplethysmography Signals

**DOI:** 10.3390/s21186241

**Published:** 2021-09-17

**Authors:** Su-Gyeong Yu, So-Eui Kim, Na Hye Kim, Kun Ha Suh, Eui Chul Lee

**Affiliations:** 1 Department of AI & Informatics, Graduate School, Sangmyung University, Hongjimun 2-Gil 20, Jongno-Gu, Seoul 03016, Korea; tnrud7495@gmail.com (S.-G.Y.); soeui291@gmail.com (S.-E.K.); nahelove03@gmail.com (N.H.K.); 2 R&D Team, Zena Inc., Seoul 04782, Korea; kunha.suh@zenacare.net; 3 Department of Human-Centered AI, Sangmyung University, Hongjimun 2-Gil 20, Jongno-Gu, Seoul 03016, Korea

**Keywords:** contact photoplethysmography, remote photoplethysmography, pulse rate variability, photoplethysmography, cardiovascular system

## Abstract

Pulse rate variability (PRV) refers to the change in the interval between pulses in the blood volume pulse (BVP) signal acquired using photoplethysmography (PPG). PRV is an indicator of the health status of an individual’s autonomic nervous system. A representative method for measuring BVP is contact PPG (CPPG). CPPG may cause discomfort to a user, because the sensor is attached to the finger for measurements. In contrast, noncontact remote PPG (RPPG) extracts BVP signals from face data using a camera without the need for a sensor. However, because the existing RPPG is a technology that extracts a single pulse rate rather than a continuous BVP signal, it is difficult to extract additional health status indicators. Therefore, in this study, PRV analysis is performed using lab-based RPPG technology that can yield continuous BVP signals. In addition, we intended to confirm that the analysis of PRV via RPPG can be performed with the same quality as analysis via CPPG. The experimental results confirmed that the temporal and frequency parameters of PRV extracted from RPPG and CPPG were similar. In terms of correlation, the PRVs of RPPG and CPPG yielded correlation coefficients between 0.98 and 1.0.

## 1. Introduction

PRV has been mainly used in recent years in the healthcare field to predict and diagnose diseases, assess stress levels, and analyze sleep stages [1,2,3,4,5,6]. The autonomic nervous system (ANS) changes according to the changes in the internal/external environment, and the changes in pulse rate (PR) caused by such changes caused by the ANS are referred to as PRV [3]. PRV is related to the interactions between the sympathetic and parasympathetic nerves that affect the sinus node and reflects transient PR and peak-to-peak interval (PPI) fluctuations. In other words, it refers to the minute variability between a specific cardiac cycle and the next [7]. The PR signal required for PRV analysis is usually acquired with electrocardiography (ECG) [8,9,10,11] or a CPPG sensor [11,12,13], which is attached to the finger. The CPPG measures the amount of light reflected by penetrating light at the finger. The amount of reflected light provides an indication of the changes in blood flow [14]. Thus, the BVP can be obtained. However, given that this CPPG sensor is not disposable, it may cause rejection in that someone who may attempt to use the sensor again may find it inconvenient to attach it. In addition, for BVP measurements, it is important to minimize motion to prevent motion artifact-induced noise, but it is difficult to keep the subject stationary with the PPG sensor attached to the finger.

Conversely, RPPG measures BVP with a camera. Thus, no sensor attachment is required. The RPPG technology extracts skin pixels from the face data captured by the camera and then uses the skin color change according to the heartbeat to obtain the BVP signal. The measurement is simple and can minimize the complexity and rejection of sensor reuse and sensor attachment. Additionally, the camera required for measurements is extensively available in the form of a webcam or a smartphone, and anyone can easily measure it. Because of these advantages, RPPGs are used in many fields such as healthcare, fitness, and forensic science. Additionally, there is focus on PRV analysis through RPPG in terms of its daily use in healthcare technology. When RPPG technology is provided as a function of a smart mirror or smartphone, it can be a great advantage to provide a more professional health indicator in addition to simply providing the average PR. Accordingly, many studies on RPPG-based PR detection and performance improvement have been reported. In addition, some RPPG-based PRV analysis studies similar to ours were also investigated.

- Verkruysse et al. [15] showed that, although the green channel generally represents the strongest plethysmography information, the blue and red channels also contain plethysmography features.
- Philipp et al. [16] explained that the periodic inflow of blood affects both the optical properties of the facial skin and head movement. In addition, based on this, a remote PR measurement technique, which use periodic changes in skin color and periodic head movements, was proposed.
- Gunther et al. [17] proposed a framework based on the use of the Markov process to account for large-scale and slowly varying fluctuations in reflected light and the quasiperiodic process to model the relatively small PR components to control the lighting and motion in RPPG technology.
- Macwan et al. [18] found that blood volume pulses produce periodic changes in skin color. These changes were quantified as a time signal and were analyzed for PR detection with the use of the RPPG by expanding the objective function of the independent component analysis.
- Song et al. [19] reported that the performance of the existing RPPG technology may be degraded owing to noise interference. Therefore, these authors proposed a method using a convolutional neural network to build a mapping between spatiotemporal PR feature images and the corresponding PR values.
- Moreno et al. [20] proposed a method for the extraction of the RR interval from the green channel component obtained from video PPG imaging and the calculation of the heart rate variability (HRV).
- McDuff et al. [21] reported that the frequency resolution limitation of existing red–green–blue (RGB) cameras can affect the measurement of detailed information about HRV. Therefore, RPPG was measured from cyan, green, and orange channel components using a new five-band camera, and an HRV analysis method using this was proposed.

However, as the existing RPPG technology extracts the pulse rate rather than the raw BVP data, it is difficult to extract health status indicators through BVP signal processing. By contrast, the lab-made RPPG technology we use can extract a single PR as well as the continuous BVP signal and a continuous PR in real time with a camera [22]. In addition, signal extraction is possible with a simple webcam set at a low sampling rate, such as 30 Hz. In addition, information such as peak, PPI, PRV, and saturation of peripheral oxygen can be obtained as health status indicators with the use of the extracted BVP signal. This allows for relatively more convenient and accurate health analysis and monitoring. Therefore, in this study, BVP signals were obtained using RPPG, which is a lab-made technique, and a PRV analysis was performed. After that, by comparing with the PRV results of CPPG measured at the same time, we intend to confirm that the PRV analysis quality using the lab-based RPPG signal is similar to the PRV analysis quality of CPPG.

The remainder of this study is as follows: Section 2 describes the lab-made RPPG technology, the experimental procedure, data processing methods, and the PRV indicators used. Section 3 describes the comparison results of the PRV indicator values extracted from the two types of PPG. A detailed analysis of the results is presented in Section 4. Finally, Section 5 presents the conclusions of this study and the expectations for future studies.

## 2. Materials and Methods

### 2.1. Principle of RPPG

**Figure 1:**
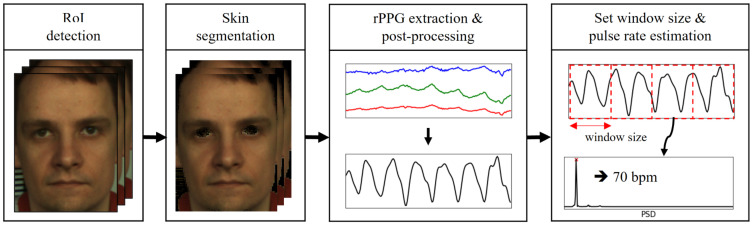
Overview of the proposed remote photoplethysmography (RPPG)-based pulse rate estimation approach.

The principle of the RPPG used to measure the BVP is as follows: Blood absorbs more light compared with surrounding tissues. Therefore, when blood passes through blood vessels, the amount of light absorbed is large, and the amount of reflected light is reduced. This causes periodic changes in skin color. RPPG is a measurement technology that uses the skin color change to measure the BVP. The RPPG acquisition process was performed as follows: First, a face was detected in an image frame using the Viola Jones algorithm, and a facial region-of-interest (ROI) was extracted using a kernelized correlation filter [23]. Subsequently, after performing skin pixel filtering using the empirically found YCbCr range of skin color, an RPPG signal, that is, a BVP signal, was obtained using a color difference-based method. The existing color difference-based method projects sequential RGB image frames on the chromaticity plane and obtains a pulse signal that is strongly correlated with the movements among them. At this time, the method encounters a problem in that the skin color change of the frame is too subtle, and the signal may be distorted owing to environmental factors. Therefore, in [22], the RGB frame extracted with the webcam is directly converted to YCbCr; skin pixel clustering is then performed on the Cb–Cr plane. Subsequently, the distribution of fine skin pixels was expanded by randomly expanding the Cb and Cr components n times from the cluster center value P (Cb-center, Cr-center). A single BVP was generated using the expanded Cb and Cr signals. Finally, to improve signal quality, breathing trends were eliminated by referring to Equation (2) proposed by de Hann et al. [24], and noise was filtered with a Butterworth bandpass filter. Based on this process, the PR can be extracted from an RGB camera at a sampling rate of 30 Hz. In addition, unlike previous studies that could measure only a single PR and not an entire signal, it is possible to continuously extract the BVP signal as well as PR [22]. This RPPG extraction process is shown in Figure 1, and the RPPG technology execution video has been published in [25].

### 2.2. Experimental Setup

PRV analysis is performed using the PPI of the BVP signal. The BVP signal measurement process required for PRV analysis is as follows. The number of subjects was 10 in total, and CPPG and RPPG were measured simultaneously. The experiment lasted for 11 min. The CPPG sensor was attached to the finger, and the face did not move away from the webcam for the RPPG measurement. Before the onset of the experiment, the subjects were instructed to minimize the movement of the fingers and face to prevent noise caused by motion noise. The CPPG sensor used was a Ubpulse 360 device with a sampling rate of 255 [26]. A Logitech general RGB webcam with a sampling rate of 30 was used for RPPG measurements [27]. The preprocessing and PPI extraction procedures for the BVP signals of CPPG and RPPG measured by these methods are described in Section 2.3 below.

### 2.3. Data Processing

**Figure 2:**
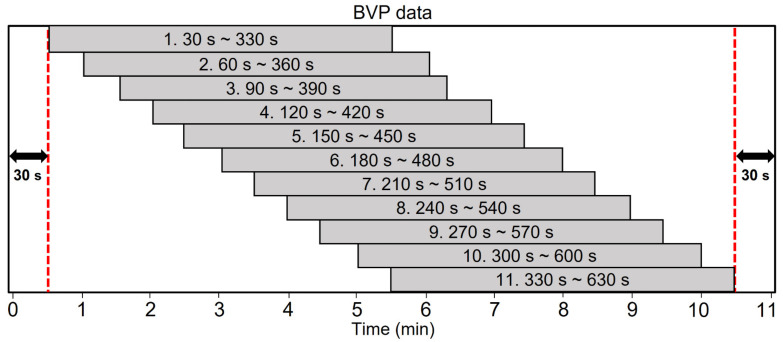
Policy for splitting 11-minute BVP data into eleven 300-second pieces of data.

**Figure 3:**
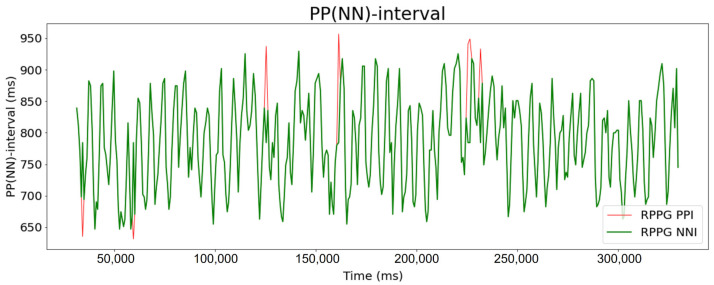
Examples of peak-to-peak interval (PPI) and normal-to-normal interval (NNI) extracted from RPPG.

First, the BVP signals obtained through the experiment (with total durations of 11 min) were modified to span a total of 10 min of data by cropping the first 30 s and the last 30 s according to considerations pertaining to the signal stability immediately before or after the experiment. To increase the number of data, 10 min of data were overlapped and divided by 300 s. The method of splitting the data by overlapping is as shown in Figure 2. As a result, 11 CPPGs and 11 RPPGs were generated for each subject. The sampling rate of RPPG was kept at 30; however, PRV analysis data with a sampling rate of 5 Hz can generally be used for calculating mean pulse rate, but a higher sampling rate is required for accurate analysis [28]. Therefore, data interpolation is performed with respect to the measured RPPG to be equal to the number of CPPG data at a sampling rate of 255. For data interpolation, a quadratic spline interpolation method that connects the data to a quadratic polynomial was used. Subsequently, bandpass filtering was performed to preserve only the 0.5 Hz to 2.0 Hz components to eliminate noise mixed with the signal [29,30]. Finally, peaks were detected in the filtered data, from which the PPI and the distance between the peaks were extracted. In addition, the PPI values with z-score values ≥ threshold T were removed to obtain more accurate PPI outcomes. The z-score indicates how far the data are from the average. T was empirically set to a value of 2, and the PPI from which the value was removed was replaced with the median value of the front and back PPIs. Through this process, the normal-to-normal interval (NNI) was finally obtained by removing the values out of the mean distribution from the PPI. Figure 3 shows an NNI extraction example based on the PPI preprocessing.

### 2.4. PRV Analysis

PRV analysis was performed using the NNI obtained through the previous process. The unit of NNI is ms. The mean NN and the standard deviation of the NNI (SDNN) were used as PRV analysis indicators for time domain analysis; low frequency (LF), high frequency (HF), and LF/HF were used for frequency domain analysis. Mean NN represents the mean of all NNIs, and SDNN represents the degree of change and responsiveness of PR as the standard deviation of all NNIs. It is an indicator that provides information related to the stability of the cardiovascular system and the ability to control the ANS and is typically used in PRV analysis. LF refers to low-frequency power data with components between 0.04 Hz and 0.15 Hz. This is an indicator related to the sympathetic nervous system and is activated when it is in a stable state. HF represents the HF power of the data, which contain components in the range 0.15–0.4 Hz and are related to the parasympathetic nervous system. At this time, the LF normalized unit (${\mathrm{LF}}_{\mathrm{nu}}$) and HF normalized unit (${\mathrm{HF}}_{\mathrm{nu}}$), which standardized the values of LF or HF power, were used because there are individual differences in LF and HF powers for each subject. This was obtained by dividing the LF (or HF) power by subtracting the very low frequency (VLF) from the total power and by multiplying by 100, as shown in Equation (1) below. Finally, the LF/HF is a balance between the sympathetic and parasympathetic nerves [31,32]. These five indicators are frequently used for PRV analysis in the time and frequency domains and intuitively indicate the changes and status of the autonomic nervous system. Therefore, the five indicators were derived for each dataset, and the PRV characteristics of CPPG and RPPG were compared.

(1) $${LF}_{nu}{\;or\;HF}_{nu}\;\left(\%\right)=\frac{absolute\;LF\;\mathrm{or}\;HF\;power}{Total\;power-VLF\;power}\;\times \;100$$

## 3. Results

The PRV results were obtained and analyzed based on the lab-made RPPG data processing and compared with CPPG. PRV analysis was conducted using the extracted NNI and PRV indicator values (mean NN, SDNN, ${\mathrm{LF}}_{\mathrm{nu}}$, $HF_{nu}$, and LF/HF) of CPPG signals, which were tested for ground-truth. The PRV similarity evaluation metrics for CPPGs and RPPGs used the mean absolute percentage error (MAPE). This is an indicator expressing similarity as a percentage of error and indicates the percentage error which occurs between the analyzed PRV value of RPPG and the analyzed PRV value of CPPG, which is the ground truth value. The closer it is to zero, the better is the regression performance. In the MAPE equation, n is the number of errors, and $x_{i}$ is the ground-truth value, which is the PRV indicator value of the CPPG. Finally, ${\hat{x}}_{i}$ indicates the PRV indicator value of the RPPG. Herein, i denotes the number of data segments among n datasets. The MAPE formula can be expressed as

(2) $$MAPE=\frac{100}{n}\overset{n}{\underset{i=1}{\sum }}\frac{\left|x_{i}-{\hat{x}}_{i}\right|}{x_{i}},$$

**Figure 4:**
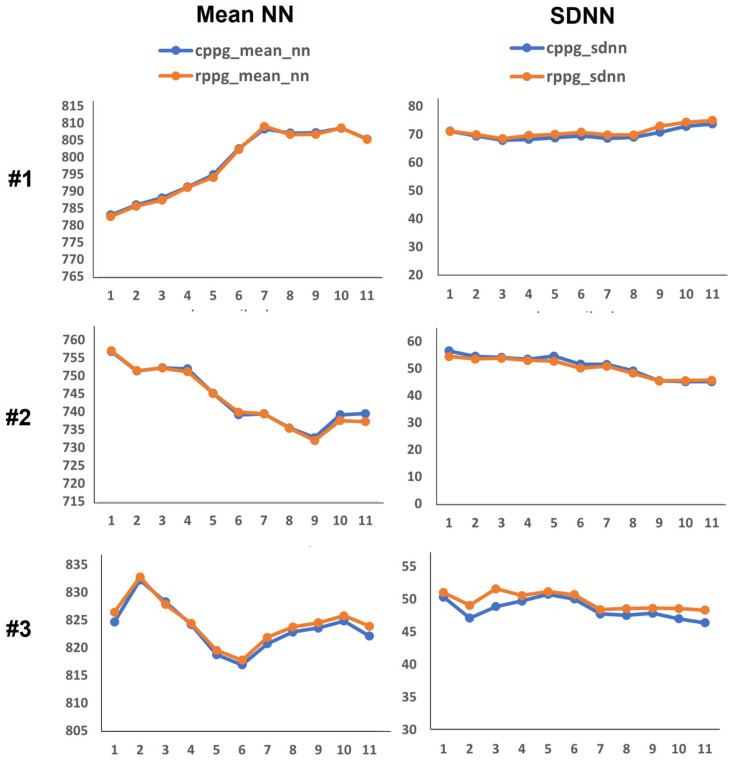

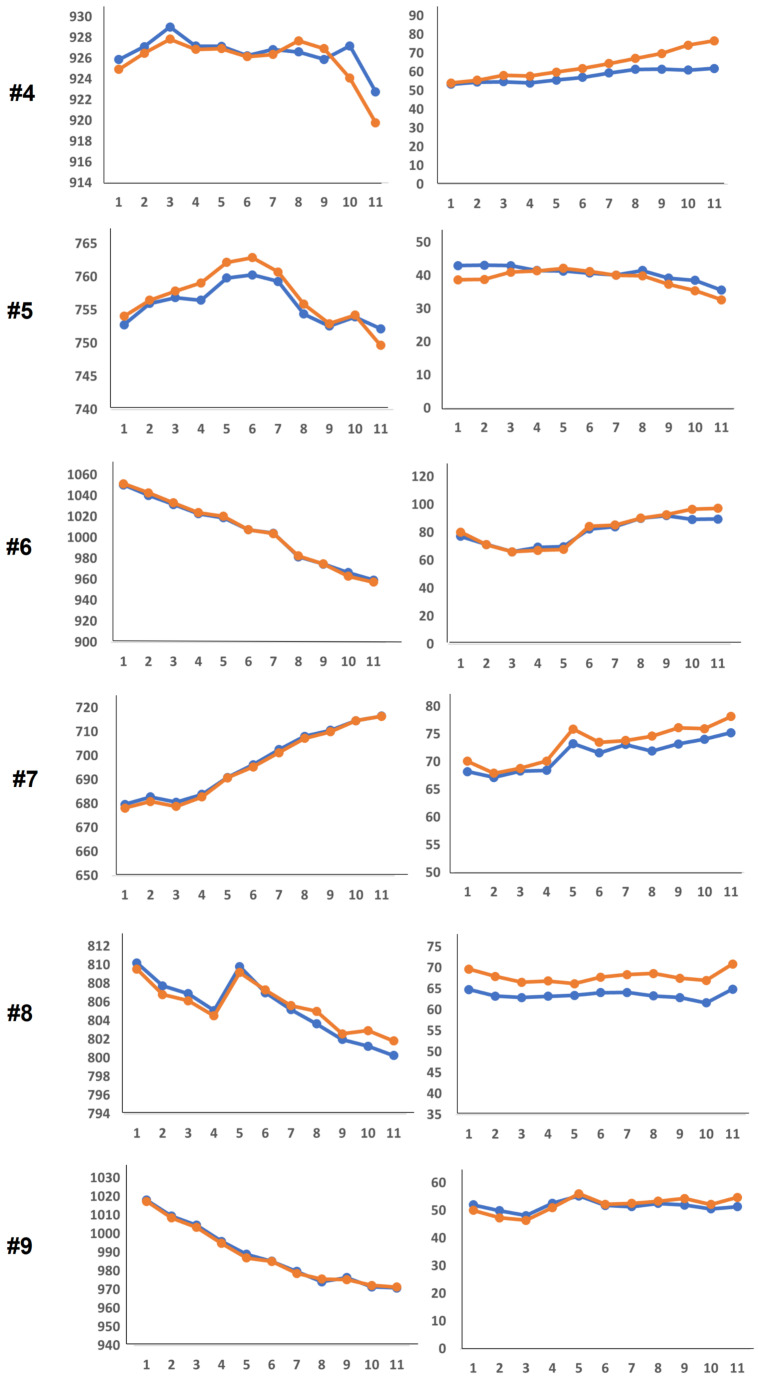

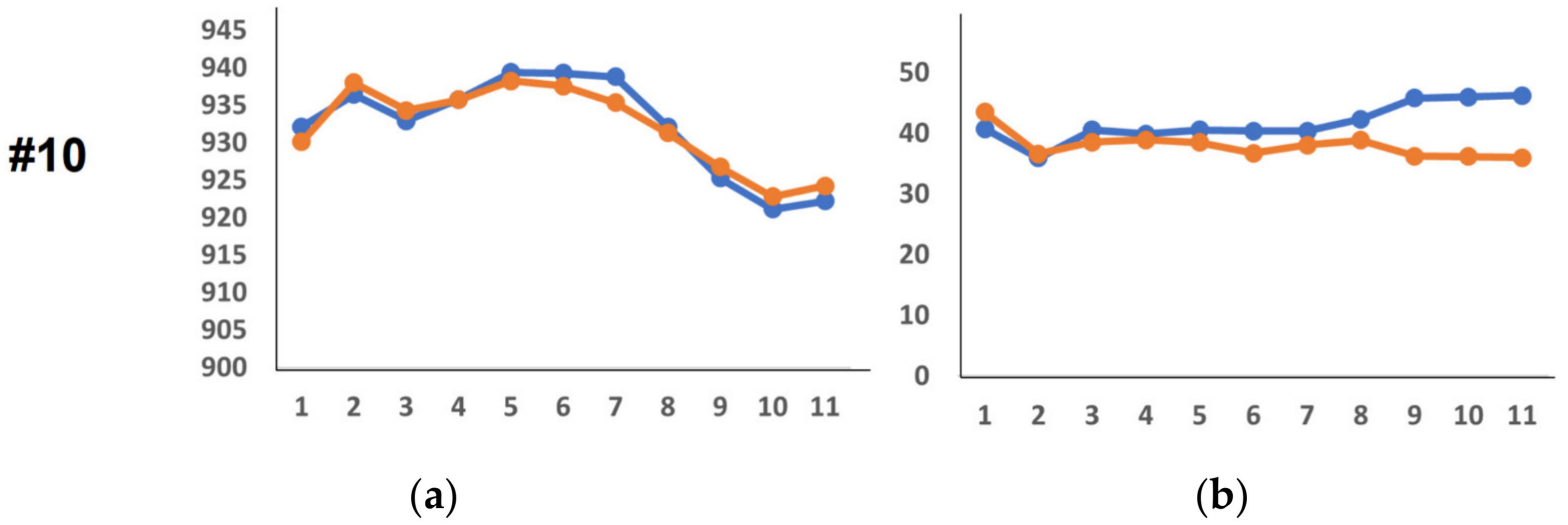
Pulse rate variability (PRV) time domain feature graphs of 10 subjects ((**a**): Mean normal-to-normal (NN), (**b**): standard deviation of NN (SDNN)).

**Figure 5:**
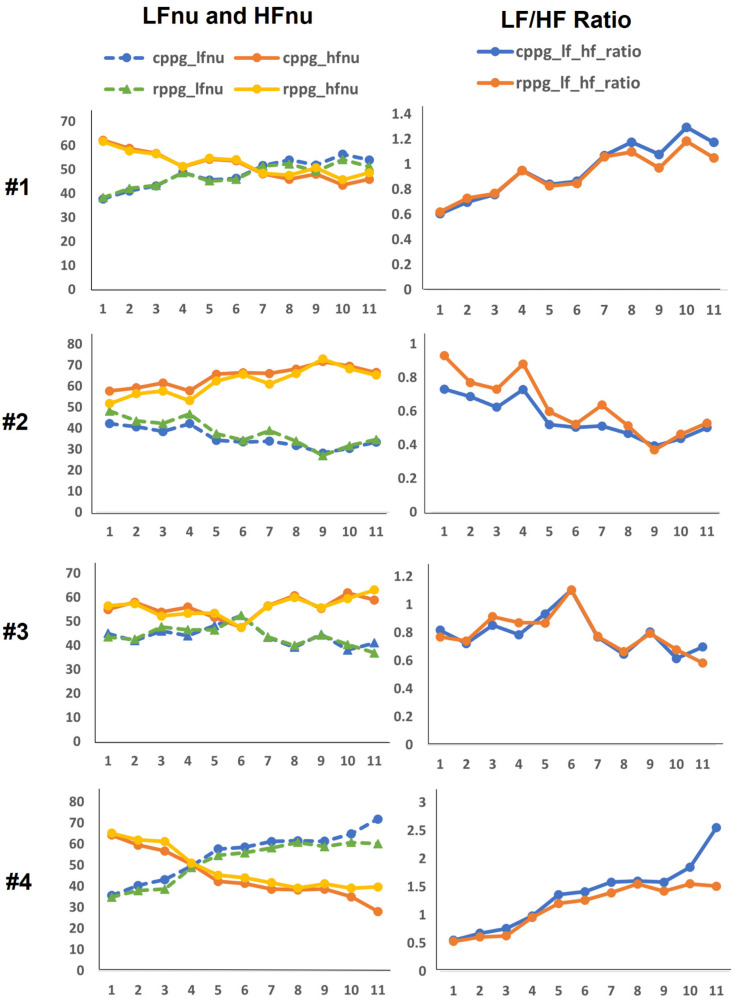

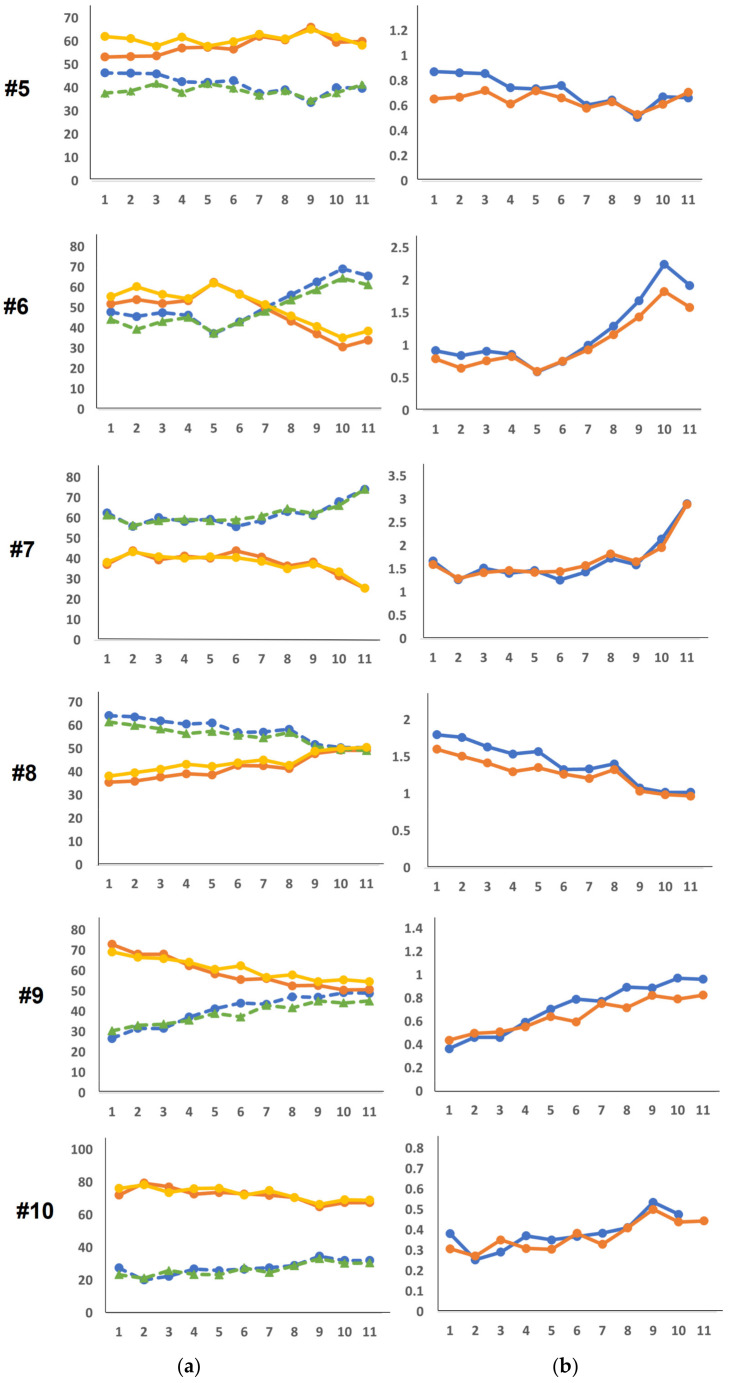
PRV frequency domain feature graphs of 10 subjects ((**a**): low frequency (LF) and high frequency (HF), (**b**): LF/HF).

**Figure 6:**
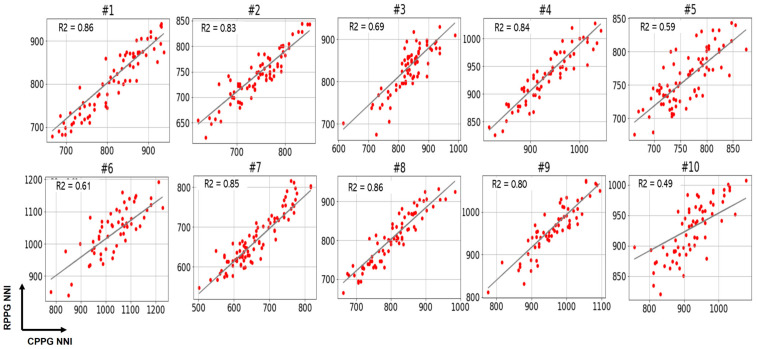
Plots for the analysis of correlations between two NNIs of contact photoplethysmography (CPPG) and remote PPG (RPPG) for 10 subjects.

The experimental results are as follows: First, Figure 4 and Figure 5 show PRV graphs in the time and frequency domains for 10 subjects. As shown in Figure 2, we have eleven data for each subject through data splitting. Accordingly, the *x*-axis of the graph plots the eleven data segments for each subject, and the vertical axis represents the value of the PRV indicator. In the graphs of the PRV indicator for each subject, the PRV values of RPPGs generally yielded similar outcomes and trends to those of CPPGs. Presently, if we analyze the LF/HF graph (Figure 5) in detail, it can be observed that there are cases wherein the LF/HF of CPPG yield increased values compared with those of RPPG. For example, in the case of the LF/HF of subject 5, it can be observed that the ratio of RPPG is smaller than that of CPPG in data segments 1 to 4 (out of 11). Therefore, observing the results of ${\mathrm{LF}}_{\mathrm{nu}}$ and ${\mathrm{HF}}_{\mathrm{nu}}$ in the corresponding section, the ${\mathrm{HF}}_{\mathrm{nu}}$ value of RPPG was measured to be larger than the ${\mathrm{HF}}_{\mathrm{nu}}$ CPPG value, and the ${\mathrm{LF}}_{\mathrm{nu}}$ value of RPPG was measured to be smaller than the ${\mathrm{LF}}_{\mathrm{nu}}$ value of CPPG. The values of LF and HF are inversely proportional to each other, thus resulting in a difference in these indicator values. Consequently, a large difference in the LF/HF occurs between the CPPG and RPPG according to a section that has a relatively small ${\mathrm{LF}}_{\mathrm{nu}}$ and a large ${\mathrm{HF}}_{\mathrm{nu}}$ in the RPPG. In Figure 6, the NNI of two PPGs of 10 subjects is plotted, and the correlation coefficient is shown on the graph. The average correlation coefficient was 0.74, and 7 out of 10 subjects had a correlation coefficient value of approximately 0.7 or higher. As shown in the graph, the NNI of the two PPGs is plotted in a straight line, so it may be expressed in the form of a linear relationship.

The error MAPE between the CPPG PRV indicator value and the RPPG PRV indicator value extracted from each of the 10 subjects are as follows: First, Table 1 shows the MAPE when using the PPI. The mean MAPE values of mean PP and SDPP were approximately 0.033% and 16.01%, respectively, and the mean MAPE values of ${\mathrm{LF}}_{\mathrm{nu}}$ and ${\mathrm{HF}}_{\mathrm{nu}}$ were approximately 10.39% and 12.59%, respectively. The LF/HF value was approximately 17.47%. Among them, subjects 3, 4, and 5 yielded relatively high MAPE values in all indicator cases except the mean PP. Therefore, NNI was obtained by performing the PPI preprocessing mentioned in Section 2.2. As indicated in Table 2, the performance was improved when PRV was analyzed with the use of NNI. Mean NN average MAPE increased to approximately 0.11%, which is a very low error rate. The mean MAPE of SDPP, ${\mathrm{LF}}_{\mathrm{nu}}$ and ${\mathrm{HF}}_{\mathrm{nu}}$ decreased to approximately 4.60%, 5.43%, and 4.96%, respectively. The mean MAPE value of LF/HF also yielded very low MAPE values which were approximately equal to 9.79%. As a result, MAPE of Mean NN(PP) decreased by 0.077% (0.033–0.11), but the MAPE values of SDNN (PP) and ${\mathrm{LF}}_{\mathrm{nu}}$ improved by 11.41% (16.01–4.60) and 4.96% (10.39–5.43), respectively. It can be observed that ${\mathrm{HF}}_{\mathrm{nu}}$ and the LF/HF improved by 7.63% (12.59–4.96) and 7.68% (17.47–9.79), respectively. In particular, the PRV MAPE of the subjects whose MAPE of $HF_{nu}$ was higher than that of $LF_{nu}$ was reduced significantly. Detailed analysis of this is performed in Section 4.

## 4. Discussion

Herein, we interpret in detail the PRV comparison results of the two PPGs. Previously, it was confirmed that normal BVP signal detection and PRV analysis using lab-based RPPG was similar to that of CPPG PRV analysis. At this time, Table 1 and Table 2 above indicated that, when PRV analysis was performed with using PPI, each indicator yielded a MAPE ≥ 20%, but when PRV analysis was performed using NNI, MAPE was significantly reduced. Therefore, in this section, we intend to perform a detailed analysis of the case where MAPE showed a rapid decrease after PPI preprocessing.

**Figure 7:**
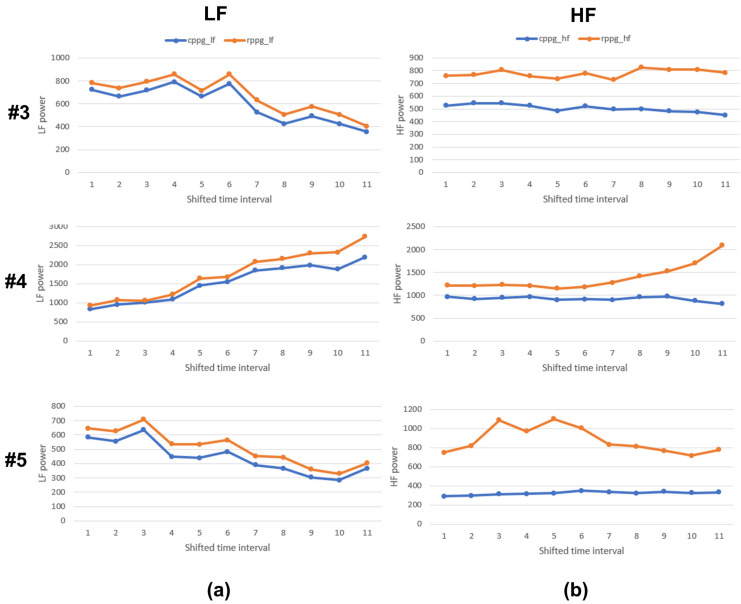
LF and HF graphs of subjects 3, 4, and 5. (**a**) LF, (**b**) HF.

In Table 1, subjects 3, 4, and 5 typically show large MAPE values in most of the PRV indicators. Their MAPE values in ${\mathrm{HF}}_{\mathrm{nu}}$ were relatively higher than in ${\mathrm{LF}}_{\mathrm{nu}}$, and the MAPEs of SDNN and LF/HF were ≥20%. The value of LF/HF is an indicator of the balance of the autonomic nervous system in the body; it is a very important PRV indicator. Therefore, for subjects 3, 4, and 5, LF and HF, which are components of the LF/HF, were examined in detail. Figure 7 shows a graph of the LF and HF of subjects 3, 4, and 5. Although there is a slight difference in the LF values of CPPG and RPPG for each subject in Figure 7a, the LF of both PPGs shows the same flows. By contrast, the HF value shown in Figure 7b shows a relatively large gap and a different flow. That is, the error between CPPG and RPPG is larger in the HF than in the LF of each subject. However, looking at Table 2, it can be observed that the MAPE of subjects 3, 4, and 5 decreased significantly. Among them, in subject 5, which had the largest MAPE, mean NN (PP) increased by 0.18%, SDNN (PP) by 35.46%, ${\mathrm{LF}}_{\mathrm{nu}}$ by 27.92%, ${\mathrm{HF}}_{\mathrm{nu}}$ by 43.48%, and the LF/HF decreased abruptly by 33.53%. Therefore, the PPI and NNI data for subject 5 were examined in detail.

**Figure 8:**
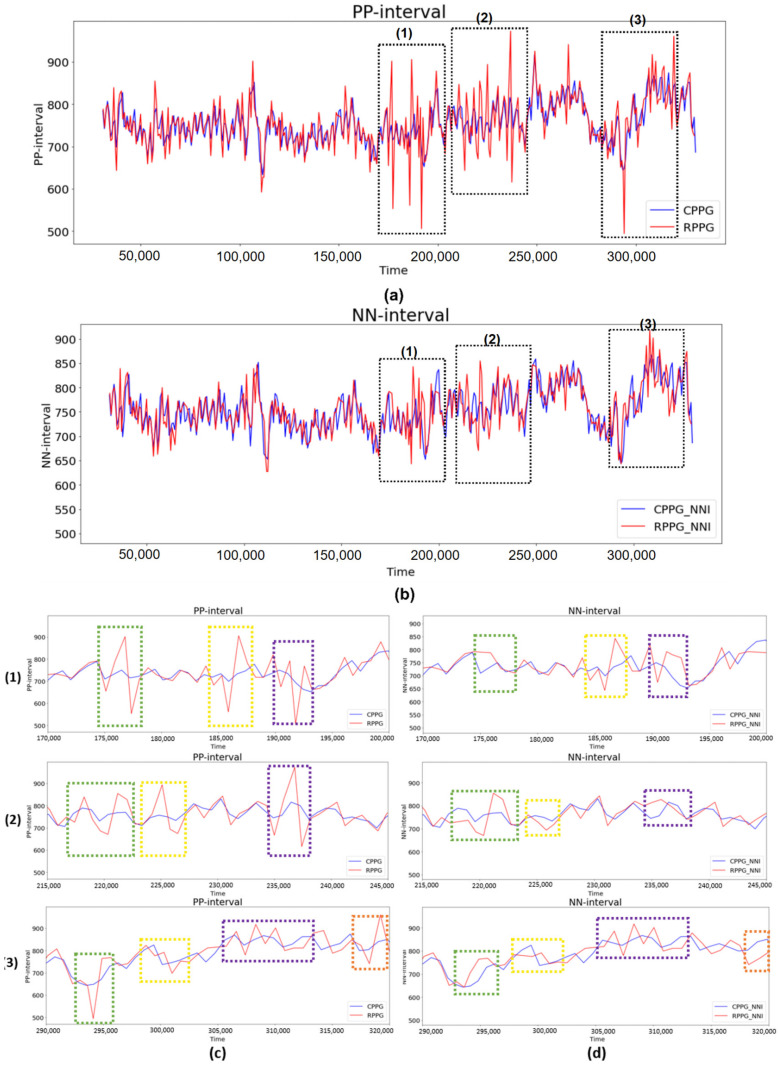
Comparison of the range ((1) to (3)) showing relatively large fluctuations in the RPPG PPI when the PPI of the RPPG and the PPI of the CPPG of subject 5 were compared. (**a**) Total PPI, (**b**) total NNI, (**c**) expanded PPI for intervals (1) to (3), and (**d**) expanded NNI for intervals (1) to (3).

**Figure 9:**
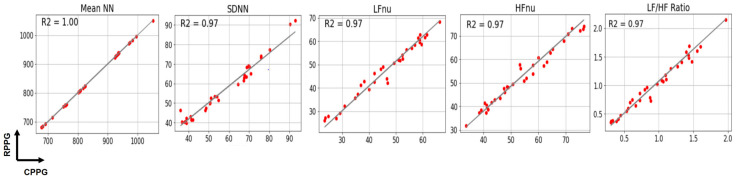
PRV indicator plots and correlation coefficients of CPPG and RPPG.

Figure 8 shows the PPI and NNI of subject 5. Firstly, the PPI graph in Figure 8a shows that the PPI of RPPG changes rapidly compared with the PPI of CPPG at (1) 170,000–200,000 ms, (2) 215,000–245,000 ms, and (3) 290,000–320,000 ms. The PPI fluctuation interval is indicated by a black dotted box in this figure. Comparing this with the NNI graph in Figure 8b, NNI shows that the signal fluctuation is reduced within the aforementioned three PPI fluctuation sections. Next, the PPI and NNI for the three PPI fluctuation sections were expanded and examined in detail. This is shown in Figure 8c,d. The magnification of the first section of 170,000–200,000 ms allowed the extraction of three detailed PPI fluctuation sections, as indicated by the green, yellow, and purple dotted line boxes. In the NNI in the same section, it can be confirmed that the NNI, which has a relatively large value, was removed and interpolated with a new value. In the second section, 215,000–245,000 ms, three additional PPI variation sections were also extracted, and the NNI of the section showed relatively reduced PPI variation. Finally, in the third section, 290,000–320,000 ms, the NNI in the section where the graph of PPI changed rapidly became more similar to the NNI of the CPPG than the PPI. In subjects 3 and 4 as well as in subject 5, a decrease in signal variability was confirmed in the NNI in the same period as the PPI time period, which showed a large difference in PPI values. It can be inferred that the observed fluctuations in PPI were caused by distortions of the BVP signal owing to the facial movements, facial expression changes (grimaces, laughter, etc.) and environmental influences (light, camera focus, etc.) during RPPG measurements. Among PRV features, HF is an indicator that is highly affected by noise generated by external factors. Therefore, in the case of subjects 3, 4, and 5, it is shown that the HF value of the RPPG was measured as various noise factors, which highly affected the accuracy of the signal during the RPPG measurement process. In addition, these noise factors seem to have affected several indicator values, such as SDNN, ${\mathrm{LF}}_{\mathrm{nu}}$, ${\mathrm{HF}}_{\mathrm{nu}}$, and LF/HF. As a result, we improved PRV analysis performance by removing and interpolating abnormal PPI values through PPI normalization, and it was confirmed that PRV analysis using lab-made RPPG is feasible at the level of PRV analysis of CPPG. Figure 9 plots the PRV indicator values using the NNI of two PPGs and shows the correlation coefficient between PRV indicator. All five indicators used show high correlation with values between 0.97 and 1.00. In the experimental results shown in Figure 7 of the previous study [21], the correlation coefficient values in $LF_{nu}$, $HF_{nu}$, and LF/HF were all >0.93. As a result of this study, the correlation coefficient values of $LF_{nu}$, $HF_{nu}$, and LF/HF were all 0.97, indicating a better correlation.

## 5. Conclusions

To confirm that PRV analysis using lab-based RPPG showed the same quality as PRV analysis using CPPG, a study was conducted to compare the PRV analysis between CPPG and lab-based RPPG. Two types of BVP signals were measured for the same subject. PRV analysis was then performed using the NNI obtained through each BVP signal processing process. Through the analysis, it was confirmed that the time and frequency domain PRV analysis using RPPG was similar to the CPPG PRV analysis based on the MAPE and correlation coefficient. In addition, based on comparison and analysis of PPI and NNI, it was confirmed that the quality of PRV analysis can be degraded owing to motion noise and various environmental factors associated with the measurement of RPPG signals. Accordingly, the accuracy of PRV analysis can be improved through additional PPI processing.

In future research, we plan to conduct a study to improve signal accuracy by developing an algorithm that can minimize noise after the analysis of each type of noise that affects the measurements of RPPG signals.

## Figures and Tables

**Table 1:** Mean absolute percentage error (MAPE) of pulse rate variability (PRV) indicators for peak-to-peak interval (PPI) for subjects studied herein.

Subject	MAPE (%)				
	Mean PP	SDPP	${\mathrm{LF}}_{\mathrm{nu}}$	${\mathrm{HF}}_{\mathrm{nu}}$	LF/HF
1	0.03	5.63	3.35	2.81	6.03
2	0.02	10.89	11.11	6.19	16.18
3	0.02	20.10	14.62	16.43	26.60
4	0.08	19.22	9.61	17.89	21.83
5	0.01	40.25	35.88	50.01	46.42
6	0.10	14.86	6.37	7.60	12.54
7	0.02	9.62	3.38	6.73	9.44
8	0.01	10.53	4.70	7.38	11.24
9	0.01	8.35	7.12	8.00	13.94
10	0.02	20.74	7.83	2.91	10.53

**Table 2:** MAPE of PRV indicators for NNI for subjects studied herein.

Subject	MAPE (%)				
	Mean PP	SDPP	${\mathrm{LF}}_{\mathrm{nu}}\;$	${\mathrm{HF}}_{\mathrm{nu}}\;$	LF/HF
1	0.05	1.58	2.20	2.32	4.36
2	0.08	1.70	7.89	4.65	13.00
3	0.11	2.50	3.41	2.56	5.94
4	0.11	9.92	5.7	8.89	12.44
5	0.19	4.79	7.96	6.53	12.89
6	0.13	2.89	5.56	6.99	11.43
7	0.13	2.58	2.20	3.40	5.71
8	0.10	6.98	3.86	5.83	9.02
9	0.09	3.24	7.74	5.57	12.65
10	0.16	9.87	7.83	2.91	10.53

## Data Availability

The obtained data cannot be shared because it was agreed that it could be used only for this study.
